# Analysis of Optimal Sensor Positions for Activity Classification and Application on a Different Data Collection Scenario

**DOI:** 10.3390/s17040774

**Published:** 2017-04-05

**Authors:** Natthapon Pannurat, Surapa Thiemjarus, Ekawit Nantajeewarawat, Isara Anantavrasilp

**Affiliations:** 1 School of Information, Computer, and Communication Technology, Sirindhorn International Institute of Technology, Thammasat University, Pathumthani 12000, Thailand; p_natthapon@yahoo.com (N.P.); ekawit@siit.tu.ac.th (E.N.); 2 National Electronics and Computer Technology Center, Pathumthani 12120, Thailand; 3 International College, King Mongkut’s Institute of Technology Ladkrabang, Bangkok 10520, Thailand; isara.an@kmitl.ac.th

**Keywords:** activity classification, activity monitoring, wearable sensors, sensor positions

## Abstract

This paper focuses on optimal sensor positioning for monitoring activities of daily living and investigates different combinations of features and models on different sensor positions, i.e., the side of the waist, front of the waist, chest, thigh, head, upper arm, wrist, and ankle. Nineteen features are extracted, and the feature importance is measured by using the Relief-F feature selection algorithm. Eight classification algorithms are evaluated on a dataset collected from young subjects and a dataset collected from elderly subjects, with two different experimental settings. To deal with different sampling rates, signals with a high data rate are down-sampled and a transformation matrix is used for aligning signals to the same coordinate system. The thigh, chest, side of the waist, and front of the waist are the best four sensor positions for the first dataset (young subjects), with average accuracy values greater than 96%. The best model obtained from the first dataset for the side of the waist is validated on the second dataset (elderly subjects). The most appropriate number of features for each sensor position is reported. The results provide a reference for building activity recognition models for different sensor positions, as well as for data acquired from different hardware platforms and subject groups.

## 1. Introduction

The Body Sensor Network (BSN) has recently been an emerging technology that provides a platform for pervasive healthcare monitoring [1]. The technology is believed to play an important part in improving the quality of life for elderly people and patients. In healthcare monitoring, wearable sensors have been employed for several applications including energy expenditure estimation [2,3,4] and analysis [5,6], fall detection [7], fall risk assessment [8], activities of daily living (ADLs) classification [9,10], motor rehabilitation [11], and cardiac monitoring [12].

Activity recognition is particularly useful in pervasive sensing systems. For fall monitoring, accurate activity classification can enhance the performance of fall detection algorithms [7]. Recognition of lying postures, e.g., supine, lying on the right side, prone, and lying on the left side, is useful for developing a system for preventing pressure ulcers [13]. Accelerometers and gyroscopes are widely used as wearable sensors for activity classification. In [14,15], seven activities, i.e., walking, sitting, standing, jogging, biking, walking upstairs, and walking downstairs, were classified using an accelerometer and a gyroscope. ADL classification using an accelerometer was reported to yield better performance than a gyroscope for all activities, except for walking upstairs and walking downstairs, and the overall recognition accuracy was not improved with the additional use of the gyroscope. Compared to a gyroscope, an accelerometer requires less power [16,17] and is thus a more suitable sensor as power constraint is one of the challenging issues in a BSN application. Other problems and requirements for the effective development of a BSN application can be found in [18]. 

**Figure 1:**
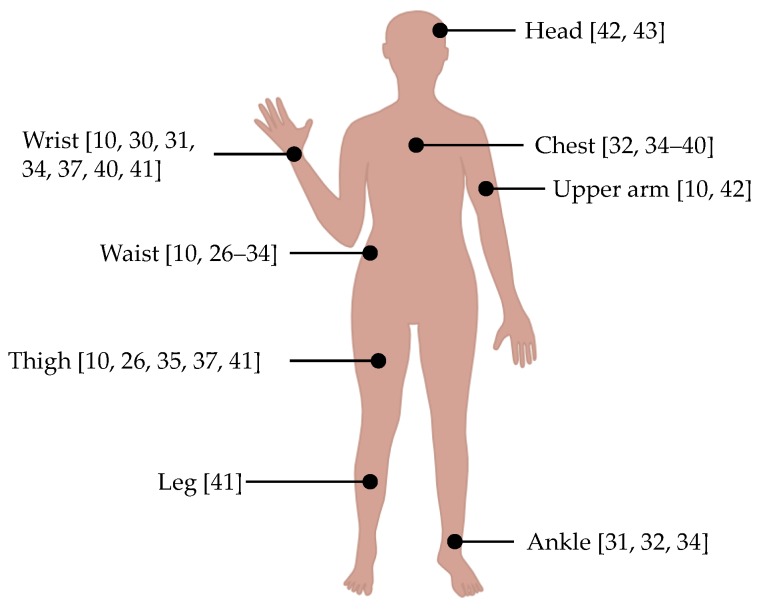
Different positions for sensor placement. Waist [10,26,27,28,29,30,31,32,33,34]; Chest [32,34,35,36,37,38,39,40]; Wrist [10,30,31,34,37,40,41]; Upper arm [10,42]; Head [42,43]; Thigh [10,26,35,37,41]; Leg [41]; Ankle [31,32,34].

In several studies [19,20,21], multiple sensors have been used either to improve detection accuracy and/or to find an optimal placement. Although higher classification accuracy has been reported with the use of multiple sensors [20], multi-sensor fusion will introduce several research challenges, as discussed in [22]. Taking usability into consideration, a smaller number of sensors is preferable. As depicted in Figure 1, a large variety of sensor positions have been examined in previous studies. In order to find an optimal sensor position, Gjoreski et al. [19] placed tri-axial accelerometers on subject’s chest, waist, thigh, and ankle. Several types of ADLs (e.g., lying, sitting, standing, sitting on the ground, sitting/lying down, standing up, and all fours on the ground) and falls (e.g., tripping, falling slowly, falling from a chair slowly, and falling from a chair quickly) were classified using statistical features, e.g., mean, root mean square, and standard deviation. With only one sensor, the accuracy values of 75% and 77% were achieved at the chest and waist, respectively. Atallah et al. [21] investigated the optimal sensor positions by placing tri-axial accelerometers on different parts of subjects’ bodies. Three feature selection algorithms, i.e., Relief, Simba, and minimum redundancy maximum relevance (mRMR), were used for measuring feature importance. These algorithms gave the same four highest ranked features. With only four features, *k* nearest neighbor (*k*NN)yielded reasonable results for distinguishing five levels of activities, i.e., very low, low, medium, high, and transactional activities. In [20], an ADL classification experiment was conducted based on five tri-axial accelerometers placed on the chest, waist, thigh, lower back, and ankle. Eleven types of features were used for classifying seven types of ADLs, i.e., lying, sitting, standing, walking, walking upstairs, walking downstairs, and jogging. Four machine-learning algorithms, i.e., decision tree (J48), naïve Bayes (NB), neural network (NN), and support vector machine (SVM), were evaluated by using a Waikato environment for knowledge analysis (WEKA) Experimenter. With a single sensor, J48 and NB yielded the best accuracy values when the sensor was placed on the ankle, while SVM appeared to be the best classifier for all other positions. Out of the four positions, the waist was reported as the best single position, with an accuracy value of 97.81%. In some studies [23,24,25], other criteria besides accuracy (e.g., computation cost, power consumption, and sensor redundancy) are also considered in the data analysis step. The experiments in these studies involved multiple sensor nodes and were beyond the scope of this study. 

Different studies focused on different types of ADLs, subject groups, hardware, and environment settings. Evaluating an algorithm on a different subject group or hardware platform usually involves collecting new data. Most of the time, the classification performances are thus not directly comparable. However, it has been learned from previous studies that a combination of features obtained from feature selection algorithms and different classification models can be used for discovering the appropriate models and features for classifying ADLs. The main objectives of this paper are twofold:

1. To investigate three different factors that affect ADL classification, i.e. sensor positions, features, and classification models.
2. To explore the possibility of applying a model trained from data collected in a different experimental setting (e.g., a subject group, a sampling rate, and hardware).

In this study, we focus on optimal sensor positioning for monitoring elderlies’ activities such as sleep postures, sitting, standing, and walking. Collecting data from elderlies (vulnerable subjects) has some limitations. They are uncomfortable with performing certain activities and/or are not able to perform certain activities for a long period of time. Walking upstairs/downstairs, for example, is not included in this study, as many of the elderlies would require assistance to perform these activities and trading the classification accuracy of walking upstairs/downstairs for that of other activities would not benefit a monitoring system for elderlies as a whole. To address the above two objectives, two datasets are used. The first dataset is collected from young subjects using tri-axial accelerometers placed on different body parts. The second dataset is collected from elderly subjects using a different hardware platform and a different sampling rate. By using the data collected from young subjects, we first study various combinations of features and models on different sensor positions and analyze how the three factors (i.e., sensor positions, features, and classification models) affect the ADL classification. The best obtained model is then applied on the dataset collected from elderly subjects. 

The paper is organized as follows; Section 2 describes the datasets used in our experiments. Section 3 presents data analysis techniques. Section 4 reports the experimental results, and Section 5 concludes this paper.

## 2. Data Descriptions

**Figure 2:**
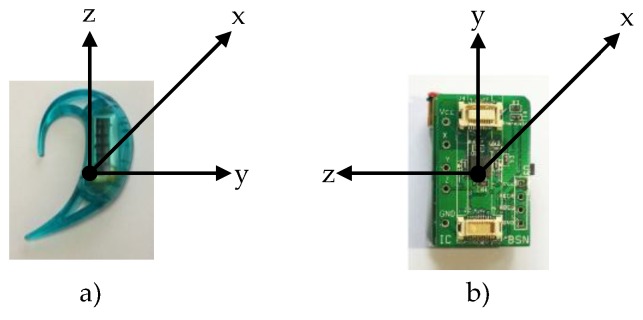
An ear-worn activity recognition (e-AR) sensor (**a**) and a Body Sensor Network (BSN) node (**b**) along with their coordinate systems.

This study involves two datasets, i.e., DS1 for deriving appropriate combinations of features and activity classification models on different sensor positions and DS2 for assessing the performance of pre-trained models when applied to data obtained using another data collection scenario. Wireless ear-worn activity recognition (e-AR) sensors [44] and a BSN node [45] developed by Imperial College were used for collecting DS1 and DS2, respectively. An e-AR sensor uses a Nordic nRF24LU1P processor with an IEEE 802.15.4 (2.4 GHz) integrated radio transceiver. A BSN node is based on a TI MSP430F1611 processor and equipped with a separate radio transceiver (Chipcon CC2420, Chipcon Co. Ltd., Oslo, Norway). Figure 2 shows an e-AR sensor and a BSN node along with the coordinate systems of their embedded tri-axial accelerometers. 

**Figure 3:**
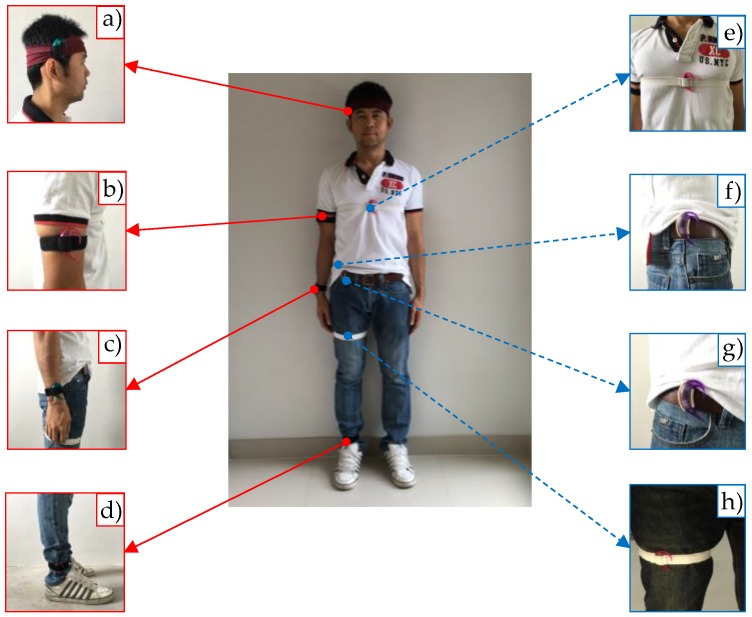
Sensor placements.

**Figure 4:**
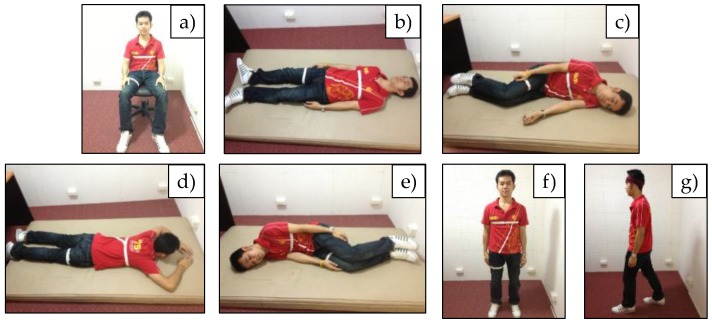
A young subject performing a sequence of seven activities.

The dataset DS1 was collected (using e-AR sensors) from 12 subjects (6 male and 6 female), aged between 23–45 years. Acceleration signals were sampled at 15 Hz and transmitted to a computer through a receiver board. As shown in Figure 3, the e-AR sensors were placed on eight different body positions, i.e., (a) the side of the head, (b) the upper arm, (c) the wrist, (d) the ankle, (e) the chest, (f) the side of the waist, (g) the front of the waist, and (h) the thigh. The twelve subjects were asked to perform a sequence of seven activities, i.e., (a) sitting on a chair, (b) supine, (c) lying on the left side, (d) prone, (e) lying on the right side, (f) standing, and (g) walking, as shown in Figure 4. Each subject performed each activity for approximately 15 s. 

**Figure 5:**
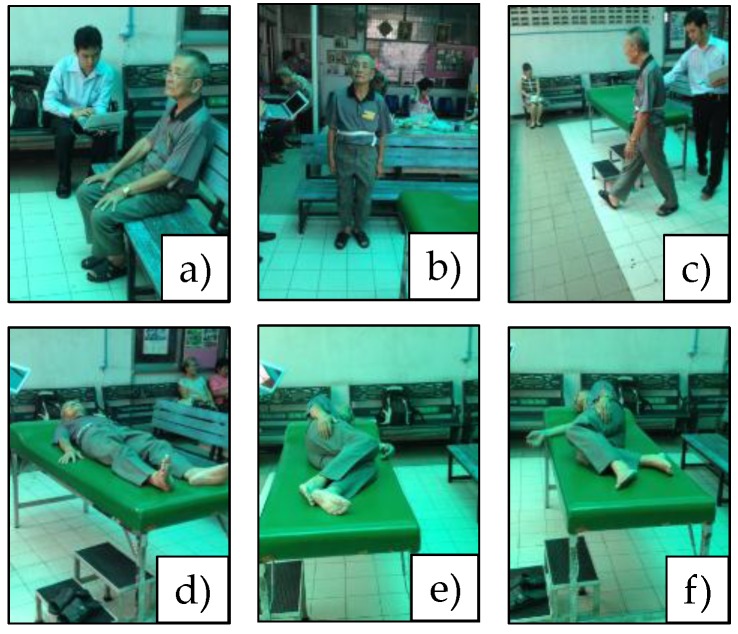
An elderly subject performing a sequence of six activities.

The dataset DS2 was collected (using a BSN node) from 48 healthy elderly subjects (20 males and 28 females), with an average age of 67.52 years. Acceleration signals were sampled at 50 Hz. The subjects were asked to perform a routine of six activities, i.e., (a) sitting on a branch, (b) standing, (c) walking, (d) supine, (e) lying on the left side, and (f) lying on the right side, which are shown in Figure 5, with a tri-axial accelerometer being attached only to the side of the waist. Compared to the activities considered in DS1, prone was excluded since it was uncomfortable for the elderly subjects. 

The study was approved by the Ethical Committee for Human Research of the National Science and Technology Development Agency (NSTDA), Thailand (document number 0010/2558), and informed consent was obtained from subjects prior to their participation.

## 3. Data Analysis

### 3.1. Feature Extraction

Prior to feature extraction, data preprocessing is required. For instance, to handle different sampling rates, acceleration signals with a high data rate were down-sampled. To cater for different device coordinate systems, a transformation matrix was used for aligning signals to the same coordinate system. To deal with noises in acceleration signals, a median filter technique was employed. To cater for inter-subject and hardware variations, acceleration signals were normalized by subtracting the median values of signals acquired during standing. Table 1 describes the features used in this study along with the feature extraction functions. The functions are applied on normalized data using a fix-sized window of 1 s, shifted by 0.5 s at each time step.

### 3.2. Feature Selection

Compared to other approaches to feature selection, a feature ranking approach, in general, requires lower computation complexity and entails a lower risk of overfitting [46]. In [21], three feature selection algorithms were examined, i.e., Relief, Simba, and mRMR, and it was reported that these algorithms yielded similar feature importance, especially for the first four highest ranked features. Relief-F [47], which is an extended version of Relief [48], was reported as the best feature selection algorithm in [49] compared to Fast Correlation Based Filter and Correlation Based Feature Selection. It was one of the most widely used feature selection algorithms, with low computational time [50] and the ability to deal with incomplete and noisy data, and can be used for evaluating feature quality in multi-class problems [51]. 

In this study, Relief-F was used to determine the most appropriate feature sets. The algorithm ranks individual features according to feature relevance scores. It randomly selects an instance *R* and finds the nearest sample *H* from the same class and the nearest sample *M* from a different class. Given a feature *A* and its feature relevance score *W*[*A*], instead of looking for the nearest sample *M* from only one different class, Relief-F searches one *M* for each different class *C* and averages their contributions to updating *W*[*A*] by

$$W[A]=W[A]-\frac{\Delta (A(R),A(H))}{n}+\sum_{c\neq class(R)}\frac{\left[P(C)\times \Delta (A(R),A(M(C)))\right]}{n},$$

where *n* is the number of instances used for approximating the probabilities; given a sample *x*, Δ(*A*(*R*), *A*(*x*)) is the difference between the value of the feature *A* of the instance *R* and the value of that of *x*; *class*(*R*) denotes the class to which *R* belongs; and *M*(*C*) and *P*(*C*) denote the nearest sample in the class *C* and the probability of the class *C*, respectively. 

### 3.3. Classification Algorithms

The following classification algorithms are used in this study.

- **Bayesian network (BN) [52]:** BN is a directed acyclic graphical model describing relationships between features and classes. Each node in a graph corresponds to a feature, and a directed edge between two nodes represents a causal relationship between them. By observing feature values and the class of each element in the set of data samples, one can construct such a network and use it to compute the probability of each class given an unseen sample. The class with the highest probability will be assigned to the sample. In our experiment, conditional probability tables are estimated by using a simple estimator, and network structures are learned from the data distribution by using the K2 search algorithm along with Bayesian scores.
- **Naïve Bayes (NB) [53]:** NB is a simple Bayes’ theorem-based probabilistic classifier with independent assumptions among features.
- **Pruned decision tree (J48) [54]:** J48 is a Java implementation of the C4.5 decision tree algorithm. C4.5 determines ‘information gain’ of each feature by comparing entropies of the data before and after considering the feature. C4.5 tries to construct a decision tree in which each node tests a feature value. Although the algorithm is proved to be very useful, features with many possible values could lead to overfitting. This problem could often be resolved by pruning some branches of the tree.
- **Partial-tree rule learning (PART) [55]:** PART uses the C4.5 decision algorithm to create a set of classification rules. However, unlike the ordinary C4.5, PART does not expand (or grow) a tree from the root to leaf nodes. It uses only a partially created tree that contains nodes with the lowest entropy to generate a set of rules. The instances covered by the created rules are then removed from the dataset. The process is repeated until all instances are covered.
- **Instance-based learning [56]:** Instead of building a classification model, an instance-based learning algorithm uses a set of given data as part of the classifier. The idea is built around an algorithm called *k* nearest neighbor (*k*NN). *k*NN treats each sample as a point in an *M*-dimensional space, where each dimension corresponds to one feature. It is assumed that elements of the same class should be close to each other (since they have similar properties, i.e. similar feature values). To classify an unseen sample, *k*NN finds *k* nearest data samples (or ‘neighbors’) and assigns the majority class of those samples to it.
- **Multi-layered perceptron** [57]**:** Sometimes referred to as neural network (NN), the algorithm classifies data samples using a layered structure (network) of small processing units, i.e., perceptrons. A perceptron takes in multiple inputs and produces a single output using a simple calculation function. Each input is associated with a computational weight. To classify a data sample, perceptrons in the first layer consider feature values of the samples and forward the output results to those in the next layer. Each perceptron in a subsequent layer produces an output by considering the results obtained from all perceptrons in its previous layer along with their corresponding weights. The process is repeated until the last layer is reached. A class is assigned to an unseen data sample based on the results of the last layer. The network can be trained to adapt itself to solve specific problems by continually adjusting the weight of each input to each perceptron.
- **Support vector machine (SVM)** [58]**:** SVM is a supervised machine learning algorithm, which can be used for both classification or regression analysis. Its basic principle is to define decision boundaries between a set of objects having different class memberships by constructing hyper planes in a multidimensional space. In our experiment, SVM is trained by applying a sequential minimal optimization algorithm with a polynomial kernel being used as a support vector.

In our experiments, we set the number *k* of neighbors for instance-based learning to 1 and 3. The performance of the eight algorithms, i.e., BN, NB, J48, PART, 1NN, 3NN, NN, and SVM, are compared. The classification models were developed in Java using WEKA application program interface with their default parameters. 

## 4. Results

### 4.1. Validation Using DS1 (Young Subjects)

**Figure 6:**
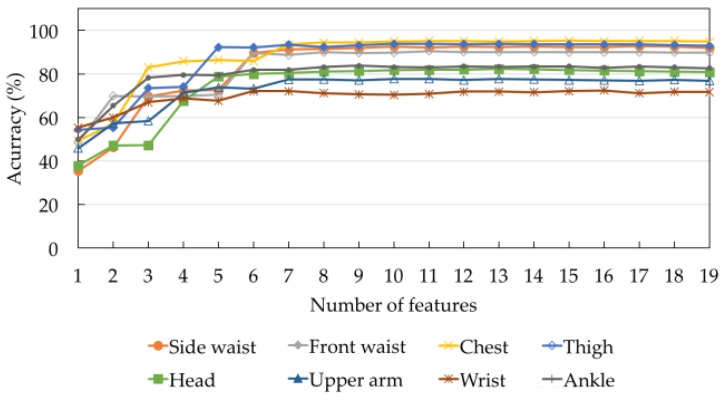
The average accuracy of the eight classification models across all sensor positions on DS1.

**Figure 7:**
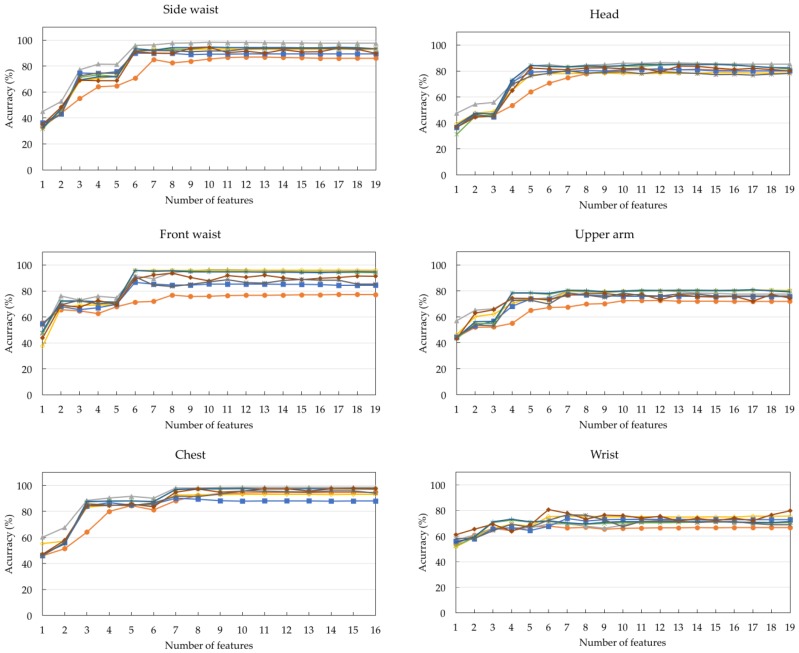

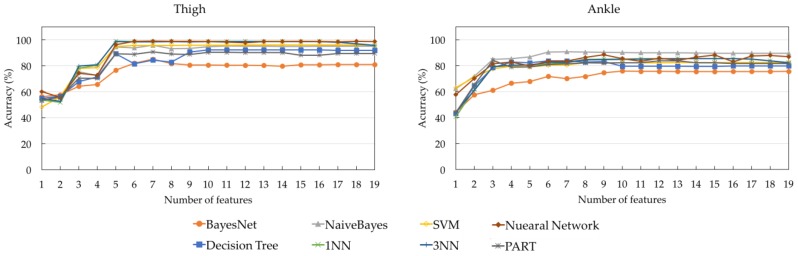
Accuracy of different classification algorithms across all sensor positions.

Table 2 shows the ranks of the 19 features obtained by applying the Relief-F feature selection algorithm to DS1. A smaller number indicates a better feature rank. The top two features used for all positions are the mean and the maximum values along the upward axis (F3 and F9). Considering the top four features, the maximum value along the x-axis (F7) and the minimum values along the upward axis (F12) are the next most commonly used features, followed by the mean along the x-axis (F1). The correlation across the three axes (F17, F18, and F19) are the three lowest ranked features for all sensor positions. Since the number of subjects in DS1 is 12, six-fold cross validation was used to evaluate the eight classification algorithms. For each fold, the models were trained based on the data acquired from ten subjects and evaluated on the data acquired from the two remaining unseen subjects. For each integer *f* such that 1 ≤ *f* ≤ 19, Figure 6 shows the average accuracy values of the eight classification algorithms across all sensor positions when the top *f* features for each sensor position were used. With only one feature, the accuracy values are relatively low for all sensor positions. The values can be improved by increasing the number of features. As there is no significant improvement on classification accuracy with further additional features, five to seven features are considered to be appropriate. The accuracy values of different classification algorithms across the eight sensor positions are shown in Figure 7. Table 3 summarizes the model settings with the best classification accuracy across the different sensor positions. The thigh yields the highest accuracy value of 99%, using 1NN with five features. NB is the best model for the side of the waist, front of the waist, chest, head, and ankle, with accuracy values of 98.34%, 96.45%, 98.50%, 86.38%, and 90.70%, respectively. 3NN and NN are the best models for the upper arm and wrist, with accuracy values of 80.83% and 80.60%, respectively.

**Figure 8:**
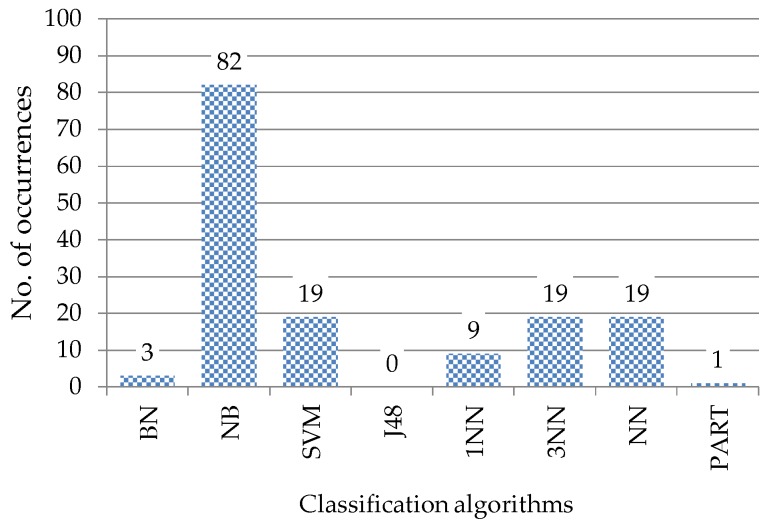
The number of occurrences of the eight classification algorithms in Table 4.

Table 4 shows the best algorithms versus different numbers of features and sensor positions. For all sensor positions except for the thigh, upper arm, and wrist, the best algorithms do not change when more than 12 features are used. It is well known that the best classification algorithm is data-dependent. The algorithm that occurs most often for a sensor position can be regarded as a generally suitable algorithm for that sensor position. From the results, NB is a generally suitable algorithm for the side of the waist, chest, head, and ankle, while 1NN, 3NN, and NN are generally suitable algorithms for the thigh, upper arm, and wrist, respectively. Apart from the front of the waist, the generally suitable algorithms are also the algorithms that yield the best classification accuracy, shown in Table 3. For the front of the waist, although SVM occurs most often as the best algorithm, it occurs only when more than the top six features are used. When a fewer number of features are used, NB is considered a generally suitable algorithm for this position. Figure 8 summarizes the frequency with which different algorithms appear as the best models across all experimental settings. In general, NB is considered the best algorithm for this dataset, followed by SVM, 3NN, and NN.

The class-specific accuracy values for the best models shown in Table 3 are detailed in Table 5. The limbs, i.e., the upper arm and wrist, are not suitable positions since they yield low accuracy for several activities (with the average accuracy being less than 85%). Average accuracy values greater than 96% are obtained from four sensor positions, i.e., the side of the waist, front of the waist, chest, and thigh. Lying postures are most difficult to classify when sensors are placed on the limbs, i.e., the upper arm, wrist, and ankle. When different subjects perform the same lying posture, their limb positions may be different. Sitting is most difficult to classify when a sensor is placed on the head; it is often misclassified as standing when a subject is sitting upright. Although the thigh appears to be the position that yields the highest average accuracy in this experiment, users may sit with different positions of their legs in realistic scenarios. In many other related works [10,21,59,60,61], a common suggestion is to place the sensor at the waist as this location is less affected by peripheral body motions than the upper or lower limbs. In terms of usability, wearing a sensor at the waist is more comfortable compared to the thigh, particularly for elderlies. 

### 4.2. Validation Using DS2 (Elderly Subjects)

**Figure 9:**
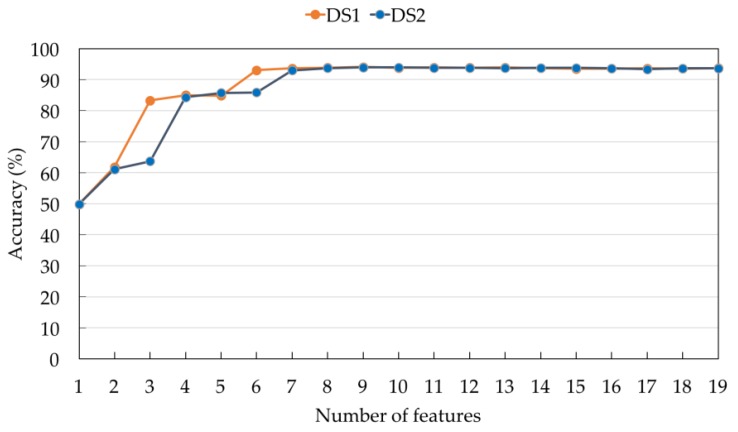
Comparison of average accuracy using feature ranking obtained from DS1 and DS2.

Feature ranking in the dataset DS2 and the possibility of applying a classifier trained from DS1 to DS2 were next investigated. Acceleration signals in DS2 were collected at a sampling rate of 50 Hz using a BSN node placed only on the side of the waist. The signals were down-sampled to approximately 15 Hz. A median filter technique was used to eliminate noise. To ensure that the features extracted from the two devices are comparable, the coordinate system of the BSN node was aligned with that of an e-AR sensor. After the matrix transformation, the Relief-F feature selection algorithm was applied to DS2. Table 6 compares the ranks of the 19 features obtained from DS1 when a sensor was placed on the side of the waist (cf. the first row of Table 2) and those obtained from DS2. At the side of the waist, the most appropriate number of features is ten (cf. Table 3). According to Table 6, all ten highest ranked features for DS1 also appear among the ten highest ranked features for DS2. Six ADL types are common to DS1 and DS2 (cf. Section 2), i.e., sitting, standing, walking, supine, lying on the left side, and lying on the right side. Figure 9 shows the average accuracy values of the eight classification models for these six common ADL types when the top *f* features were used for each integer *f* such that 1 ≤ *f* ≤ 19. Except for the case when the top three and the top six features are used, the average accuracy values on DS1 and DS2 are almost the same. Based on Table 6 and Figure 9, it is expected that the top ten features used in the best model at the side of the waist for DS1 are also appropriate for DS2. 

The best model at the side of the waist for DS1, i.e., NB, is validated on DS2 by using the top ten features derived from DS1 for the side of the waist. Table 7 shows the ADL classification results. The accuracy values of all ADL types are greater than 92%. The average accuracy is 95.77%, which is only slightly lower than the average accuracy obtained on DS1 using the same model and features (98.34%, cf. the first row of Table 3). 

### 4.3. Comparison with a Transfer Learning Method

A transfer learning was defined as the ability to extend what has been learned in one context to new contexts [62]. A summary of several existing works on activity recognition using transfer learning can be found in [63]. A recent work that is closely related to our study is presented in [64], in which three different asynchronous data mapping (ADM) algorithms, i.e., brute force ADM (BFADM), clustering-based ADM (CADM), and motif-based ADM (MADM), were introduced for supporting knowledge transfer in wearable systems. Tri-axial accelerometers (embedded in smartphones) with four different sampling rates, i.e., 50, 100, 150, and 200 Hz, were used for activity recognition. Nine young subjects were asked to perform six types of ADLs, i.e., walking, sitting, standing, walking downstairs, walking upstairs, and biking, while wearing eight smartphones (two phones for each sampling rate) on their waists. Three experiments were conducted, i.e., inter-subject, inter-device, and inter-model. The inter-subject experiment used data collected from eight subjects to train classification models and evaluated them with the remaining subject. The inter-device and inter-model experiments used data collected from one phone to train models, which were then tested using signals from another phone with the same sampling rate and signals from another phone with a different sampling rate, respectively. The classification models were trained and tested based on normalized cross-correlation with the resulting values lying between −1 and 1. Three classification algorithms were used, i.e., *k*NN, decision tree, and random forest. Among the three mapping algorithms, BFADM yielded the highest accuracy using random forest. It was concluded in [64] that the recognition accuracy of their approach could be affected by sampling frequency variation, device variation, and subject variation. In addition, cross-correlation is a poor measure to capture subject variation. Another limitation of their approach is the ability to handle static activities, especially when device orientations are different (since it relies on signal motifs, which are subsequences that occur repeatedly in time series). In our study, we use signal resampling to handle the difference in sampling frequency, data normalization to handle inter-subject variation, and data transformation to handle the difference in sensor orientations. Table 8 compares the experimental settings and classification accuracy in our study with those in [64].

## 5. Conclusions

This study presents an analysis of the optimal settings for activity classification in terms of sensor positions, features, and classifiers and assesses the possibility of applying a trained model to data acquired from a different experimental scenario. Focusing on monitoring basic activities performed by elderlies, only four lying postures, sitting, standing, and walking, are considered. 

In the first experiment, activity classification was performed on a dataset collected from young subjects using e-AR sensors attached to eight different parts of the subjects’ bodies, i.e., the side of the waist, front of the waist, chest, thigh, head, upper arm, wrist, and ankle. Nineteen features were extracted and their importance was measured by using the Relief-F feature selection algorithm. Different combinations of features and eight classification algorithms on different sensor positions were investigated. For each sensor position, the best classification model and the most appropriate features were reported. The mean and the maximum values along the upward axis were the top two common features used for all sensor positions. Among the eight positions, the side of the waist, front of the waist, chest, and thigh were the optimal sensor positions. NB outperforms other classifiers for most feature combinations. The NB algorithm with ten to eleven features was the best model for the side of the waist, front of the waist, and chest, while the 1NN algorithm with five features was the best model for the thigh.

In the second experiment, the dataset was collected from the elderly subjects using a BSN node placed on the side of the waist. To process the signals from different environment settings, signals with high sampling rates were down-sampled and coordinate systems were aligned to the same direction. The experimental results show that the best model derived from the young-subject dataset can still perform with high classification accuracy when applied to the elderly-subject dataset. This demonstrates that data collection effort could be saved when a new hardware platform is developed, i.e., classification models obtained from a previous data collection scenario could be applicable on a new hardware platform, provided that appropriate data preprocessing has been performed.

## Figures and Tables

**Table 1:** List of features and their equations.

Feature	Description	Equation
F1–F3	Means along x-, y-, and z-axes	$\mu =\frac{\sum_{i=1}^{N}x_{i}}{N}$
F4–F6	Standard deviations along x-, y-, and z-axes	$\sigma =\sqrt{\frac{\sum_{i=1}^{N}{{(x}_{i}-\mu )}^{2}}{N}}$
F7–F9	Maximum values along x-, y-, and z-axes	$M=\max(x_{i})$
F10–F12	Minimum value along x-, y-, and z-axes	$m=\min(x_{i})$
F13–F15	Differences between maximum and minimum values along x-, y-, and z-axes	Δm=M−m
F16	Standard deviation magnitude	$\left|\sigma \right|=\sqrt{\sigma_{x}^{2}+\sigma_{y}^{2}+\sigma_{z}^{2}}$
F17–F19	Correlation between x-y, x-z, and y-z axes	$r_{ab}=\frac{\sigma_{ab}}{\sigma_{a}\sigma_{b}}$

*N* = number of data samples; *i* = data sample index; $x_{i}$ = observation vector at *i*; $\sigma_{x},\sigma_{y},\sigma_{z}$ are standard deviation values along the x-, y-, and z-axes, respectively; $\sigma_{ab}$ is the covariance between axes *a* and *b*.

**Table 2:** The feature rankings obtained from Relief-F on Dataset 1 (DS1).

Position	Feature Ranks																		
	1	2	3	4	5	6	7	8	9	10	11	12	13	14	15	16	17	18	19
Side waist	F3	F9	F7	F10	F1	F11	F2	F8	F12	F16	F4	F5	F14	F15	F6	F13	F18	F19	F17
Front waist	F3	F7	F1	F9	F12	F11	F10	F2	F8	F16	F6	F15	F4	F14	F13	F5	F19	F17	F18
Chest	F3	F9	F8	F11	F2	F12	F7	F1	F10	F14	F5	F15	F6	F16	F13	F4	F18	F17	F19
Thigh	F9	F3	F1	F7	F2	F8	F11	F10	F12	F16	F4	F13	F5	F6	F15	F14	F17	F19	F18
Head	F3	F9	F12	F2	F7	F8	F11	F1	F10	F6	F15	F16	F4	F13	F14	F5	F17	F18	F19
Upper arm	F3	F9	F12	F2	F11	F8	F1	F7	F10	F16	F6	F13	F15	F4	F14	F5	F17	F18	F19
Wrist	F3	F9	F7	F12	F1	F2	F11	F10	F8	F16	F6	F15	F4	F13	F5	F14	F17	F18	F19
Ankle	F9	F3	F1	F12	F7	F2	F10	F11	F8	F16	F6	F15	F4	F5	F13	F14	F18	F19	F17

**Table 3:** Settings with the highest accuracy for different sensor positions.

Position	Number of Features	Best Algorithm	Accuracy
Side waist	10	NB	98.34
Front waist	11	NB	96.45
Chest	10	NB	98.50
Thigh	5	1NN	99.00
Head	12	NB	86.38
Upper arm	17	3NN	80.83
Wrist	6	NN	80.60
Ankle	7	NB	90.70

**Table 4:** Best algorithms for different numbers of features and sensor positions.

Position	Feature Ranks																		
	1	2	3	4	5	6	7	8	9	10	11	12	13	14	15	16	17	18	19
Side waist	NB	NB	NB	NB	NB	NB	NB	NB	NB	NB	NB	NB	NB	NB	NB	NB	NB	NB	NB
Front waist	BN	NB	NB	NB	NN	3NN	SVM	SVM	SVM	NB	NB	NB	SVM	SVM	SVM	SVM	SVM	SVM	SVM
Chest	NB	NB	NB	NB	NB	NB	NB	NB	NB	NB	NB	NB	NB	NB	NB	NB	NB	NB	NB
Thigh	BN	BN	3NN	3NN	NN	NN	1NN	NN	NN	1NN	1NN	1NN	1NN	1NN	1NN	1NN	NN	NN	NN
Head	NB	NB	NB	3NN	1NN	NB	NB	NB	NN	NB	NB	NB	NB	NB	NB	NB	NB	NB	NB
Upper arm	NB	NB	NB	3NN	NN	3NN	3NN	3NN	SVM	3NN	3NN	3NN	3NN	3NN	3NN	3NN	3NN	SVM	SVM
Wrist	NN	NN	3NN	3NN	3NN	NN	NN	PART	NN	NN	SVM	NN	SVM	SVM	SVM	SVM	SVM	NN	NN
Ankle	NB	NB	NB	NB	NB	NB	NB	NB	NB	NB	NB	NB	NB	NB	NB	NB	NB	NB	NB

**Table 5:** The activities of daily living (ADL) classification results obtained from the best models on DS1.

Position	Activities							Average
	Sitting	Supine	Lying Left	Prone	Lying Right	Standing	Walking	
Side waist	94.05	99.79	100.00	97.50	99.79	97.27	100.00	98.34
Front waist	93.63	99.66	94.06	98.61	90.48	98.69	100.00	96.45
Chest	92.72	100.00	100.00	100.00	99.83	97.14	99.81	98.50
Thigh	93.97	99.28	100.00	100.00	100.00	100.00	99.78	99.00
Head	35.20	99.05	99.18	89.09	94.50	88.40	99.26	86.38
Upper arm	92.59	71.65	84.90	49.79	69.30	98.50	99.11	80.83
Wrist	77.25	78.24	72.59	58.42	80.45	98.45	98.77	80.60
Ankle	97.61	79.91	87.71	80.31	93.42	98.82	97.13	90.70

**Table 6:** The feature rankings obtained from Relief-F on DS1 and Dataset 2 (DS2) for the side of the waist.

Dataset	Feature Ranks																		
	1	2	3	4	5	6	7	8	9	10	11	12	13	14	15	16	17	18	19
DS1	F3	F9	F7	F10	F1	F11	F2	F8	F12	F16	F4	F5	F14	F15	F6	F13	F18	F19	F17
DS2	F3	F12	F9	F10	F7	F1	F11	F2	F8	F16	F14	F5	F13	F4	F15	F6	F19	F18	F17

**Table 7:** The ADL classification results on DS2 using Naïve Bayes (NB) with the top ten features derived from DS1.

Activities						Average
Sitting	Standing	Walking	Supine	Ling Left	Lying Right	
96.03	96.78	100.00	94.32	92.04	95.43	95.77

**Table 8:** Comparison between our work and the work presented in [64].

	Our Study	Saeedi et al.’s Study
No. of subjects	12 young subjects	9 young subjects
	48 elderly subjects	
Sensor	3D accelerometers	3D accelerometers
Sampling rate	15 and 50 Hz	50, 100, 150, and 200 Hz
Features	19 features, with Relief-F feature selection algorithm	Signal similarity
Window size	1 s (shifted by 0.5 s)	2 s (shifted by 0.5 s)
Sensor placements	Side waist, front waist, chest, thigh, head, upper arm, wrist, and ankle	Waist
Activities	Sitting, supine, lying on the left side, prone, lying on the right side, standing, and walking	Walking, sitting, standing, walking downstairs, walking upstairs, and biking
Classifiers	BN, NB, J48, PART, *k*NN, NN, and SVM	*k*NN, decision tree, and random forest
Accuracy	98.34% (side waist, using NB with 10 features)	~85% (with random forest)

## Abbreviations

The following abbreviations are used in this manuscript:

BSN	Body sensor network
ADL	Activity of daily living
BN	Bayesian network
NB	Naïve Bayes
PART	Partial-tree rule learning
*k*NN	*k* nearest neighbors
NN	Neural network
SVM	Support vector machine
